# Metabolic Adaptions/Reprogramming in Islet Beta-Cells in Response to Physiological Stimulators—What Are the Consequences

**DOI:** 10.3390/antiox11010108

**Published:** 2022-01-04

**Authors:** Philip Newsholme, Jordan Rowlands, Roselyn Rose’Meyer, Vinicius Cruzat

**Affiliations:** 1 Curtin Medical School and CHIRI, Curtin University, Perth, WA 6845, Australia; 2 School of Pharmacy and Medical Sciences, Griffith University, Gold Coast, QLD 4222, Australia; r.rosemeyer@griffith.edu.au; 3 Faculty of Health, Torrens University Australia, Brisbane, QLD 4006, Australia; vinicius.cruzat@torrens.edu.au

**Keywords:** insulin, metabolic reprogramming, antidiabetic therapeutics, glucose metabolism, lipid metabolism, islet inflammation

## Abstract

Irreversible pancreatic β-cell damage may be a result of chronic exposure to supraphysiological glucose or lipid concentrations or chronic exposure to therapeutic anti-diabetic drugs. The β-cells are able to respond to blood glucose in a narrow concentration range and release insulin in response, following activation of metabolic pathways such as glycolysis and the TCA cycle. The β-cell cannot protect itself from glucose toxicity by blocking glucose uptake, but indeed relies on alternative metabolic protection mechanisms to avoid dysfunction and death. Alteration of normal metabolic pathway function occurs as a counter regulatory response to high nutrient, inflammatory factor, hormone or therapeutic drug concentrations. Metabolic reprogramming is a term widely used to describe a change in regulation of various metabolic enzymes and transporters, usually associated with cell growth and proliferation and may involve reshaping epigenetic responses, in particular the acetylation and methylation of histone proteins and DNA. Other metabolic modifications such as Malonylation, Succinylation, Hydroxybutyrylation, ADP-ribosylation, and Lactylation, may impact regulatory processes, many of which need to be investigated in detail to contribute to current advances in metabolism. By describing multiple mechanisms of metabolic adaption that are available to the β-cell across its lifespan, we hope to identify sites for metabolic reprogramming mechanisms, most of which are incompletely described or understood. Many of these mechanisms are related to prominent antioxidant responses. Here, we have attempted to describe the key β-cell metabolic adaptions and changes which are required for survival and function in various physiological, pathological and pharmacological conditions.

## 1. Introduction

**Figure 1:**
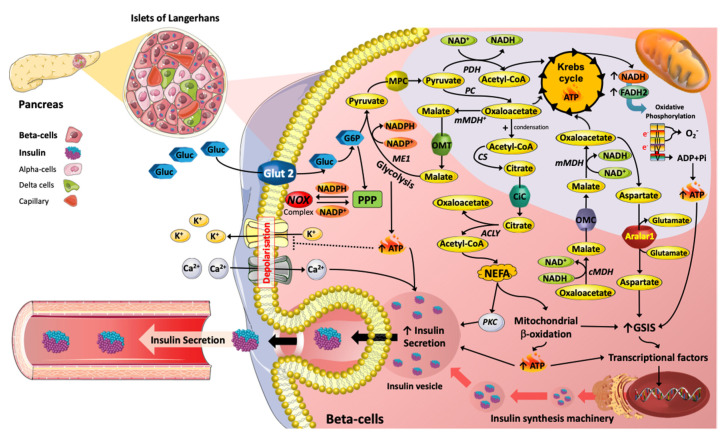
Metabolic reprograming mediated by nutrients with respect to insulin secretion machinery in Islet β cells. The metabolic pathways induced by glucose (Gluc) and lipids (e.g., NEFA) are key in the promotion of insulin secretion. For instance, TCA (tricarboxylic acid) cycle intermediates, such as Acetyl-CoA, Oxaloacetate, Malate and Citrate are essential in promoting insulin exocytosis. Moreover, TCA cycle intermediates can act as precursors for lipid signalling molecules that stimulate insulin vesicle trafficking and calcium influx, contributing to insulin secretion. Hence, changes in plasma glucose and NEFAs over physiological concentration ranges are essential for the regulation of β-cell function.

Insulin release from the pancreatic β-cell is a tightly controlled process and many factors are required to stimulate insulin release [1]. Glucose, usually derived from dietary carbohydrate, is the primary insulin secretagogue [2], as there is tight coupling between its metabolism and insulin exocytosis mainly through increases in the β-cell intracellular ATP:ADP ratio due to enhanced flux through glycolysis and the tricarboxylic acid cycle (TCA), resulting in elevated glycolytic and mitochondrial ATP generation, respectively [3]. The enhanced ATP:ADP ratio results in closure of β-cell K^+^_ATP_-sensitive channels and subsequent Ca^2+^ influx through voltage-gated calcium channels (Figure 1). An increase in both intracellular ATP and Ca^2+^ levels leads to fusion of readily releasable pools of insulin-containing vesicles with the plasma membrane [1,4]. This *triggering* mechanism of K_ATP_-dependent GSIS is responsible for ‘first phase’ insulin secretion, over the first 5–10 min of stimulation. The more sustained second phase of insulin release occurring over 30–60 min (*amplifying pathway*) is absolutely dependent on continued closure of β-cell K^+^_ATP_-sensitive channels, mitochondrial metabolic activity and generation of stimulus–secretion coupling factors, initially described in rodent β-cells in 1992 [5,6]. Thus, the impact of specific nutrient groups and associated nutrient excesses on pancreatic β-cell function and dysfunction including insulin secretion will be discussed, along with immune-mediated, incretin-mediated and pharmacological drug-mediated metabolic adaptions/reprogramming. It is now appreciated that cells can undergo regulated reprogramming of their metabolic capacities to acquire new cellular and biological functions. An example of such reprogramming, as described by Otto Warburg, is a large shift in glycolytic metabolism and reduction in oxidative metabolism in tumor cells. The main metabolic pathways in the β-cell responsible for the regulation of insulin secretion have recently been reviewed in detail [7]. While evidence for metabolic reprogramming and associated adaptions have been described in detail for some cells including cardiomyocytes, cancer cells and macrophages [8,9,10,11,12], as yet, full appreciation of the various metabolic adaptions in the β-cell is not evident.

## 2. Evidence for Nutrient Regulation of Metabolic Adaptions/Reprogramming in β-Cells

### 2.1. Glucose

If K^+^_ATP_-sensitive channels are prevented from closing by addition of diazoxide [6,13] in the presence of high concentrations of K^+^ (to depolarize the plasma membrane) and glucose, insulin release will occur. Additionally, in mice with genetically disrupted K^+^ channels insulin release was still possible, thus there is an additional nutrient-stimulated secretory mechanism that regulates sustained insulin release [14,15]. This mechanism is known as K^+^_ATP_-independent GSIS, it is dependent on levels of TCA intermediates and associated products (anaplerosis), phospholipase C/protein kinase C (PKC) signalling, various lipids and/or elevation in cyclic adenosine monophosphate (cAMP) levels, thus elevating cytosolic Ca^2+^ flux and exocytosis (Figure 1) [16,17]. Known coupling factors, which can amplify K_ATP_-independent GSIS, include NADPH, NADH, glutamate and malonyl-CoA [1,18]. Therefore, β-cell nutrient metabolism is central and critical to insulin secretion. However, chronically elevated glucose and lipid levels (associated with T2DM) can reduce insulin secretion.

Chronically elevated glucose concentrations or indeed amino acids such as alanine can desensitize the β-cell when subjected to subsequent acute challenge with the same nutrient [19]. A recent report [20] has detailed that desensitization of mouse islet cells to glucose is probably related to increased Ca^2+^ influx in the cytosol and subsequently mitochondria (via activation of Ca^2+^ transporters in the mitochondrial membrane), depletion of TCA cycle intermediates and reduction in production of metabolic stimulus–secretion coupling factors including ATP after an initial burst. In addition, ATP consuming Ca^2+^ exporting ATPases in the plasma membrane further depleted ATP levels. The desensitization could be removed by experimental activation of glutamate dehydrogenase or pyruvate carboxylase (PC), thus increasing TCA cycle intermediates via anaplerotic mechanisms involving generation of alpha-ketoglutarate or oxaloacetate, respectively, thus partially restoring ATP synthesis and generation of mitochondria derived metabolic stimulus–secretion coupling factors.

Pancreatic β-cells express low levels of LDHA (encoded by a ‘disallowed’ gene, [21]) but compensate by higher mitochondrial redox shuttle flux (generating NADH/NADPH) including the pyruvate/malate and pyruvate/citrate shuttles [1,22]. An essential component of pyruvate-dependent shuttles is generation of oxaloacetate from pyruvate (Figure 1). Oxaloacetate is then converted to malate and exported to the cytosol. Malic enzyme 1 (ME1) can regenerate pyruvate from malate but additionally generates NADPH (Figure 1). Pyruvate can re-enter the mitochondria, and will be converted to oxaloacetate, thus continuing the process. Citrate may alternatively be formed from oxaloacetate and acetyl-CoA via citrate synthase and then transferred to the cytosol via the citrate carrier (Figure 1). Citrate may be metabolised to oxaloacetate and acetyl CoA via ATP Citrate Lyase, Oxaloacetate then converted to malate and allowing ME1 to generate NADPH as described above, while acetyl CoA promotes LC-acyl CoA synthesis (dependent on acetyl CoA carboxylase, ACC) via malonyl-CoA formation. LC-acyl CoA subsequently promotes insulin secretion [23,24] (Figure 1). Another vital redox shuttle in β-cells is the malate/aspartate shuttle (Figure 1). Oxaloacetate can be converted to malate and NAD^+^, via MDH, and malate can then enter the mitochondrion and subsequently can be metabolised to oxaloacetate by mitochondrial MDH (mMDH), resulting in NAD^+^ reduction to NADH (Figure 1). Mitochondrial oxaloacetate can be transaminated to aspartate and then transported to the cytosol through the aspartate/glutamate carrier, Aralar1 [25,26]. Deletion of Aralar-1 in INS-1 β-cells resulted in loss of malate/aspartate shuttle activity and importantly, a substantial decrease in insulin secretion, of around 25% [26]. In addition, increased expression of Aralar1 enhanced GSIS and improved insulin secretion promoted by amino acids in BRIN-BD11 β-cells [27]. The impacts of type-2 diabetes mellitus (T2DM) on the expression of carriers and enzymes described above are not well defined or described. These critical metabolic regulatory components are probably altered in both expression and activity levels as part of adaption/reprogramming mechanisms. This requires further work on islets.

Abbreviations: ACLY, ATP citrate synthase (also known as ATP citrate lyase); ADP, Adenosine di-phosphate; ATP, Adenine triphosphate; Ca^2+^, Calcium; CiC, Citrate transporter; cMDH, Cytosolic malate dehydrogenase; CS, Citrate synthase; FADH, Flavin adenine dinucleotide reduced Hydrogen; G6P, Glucose 6-phosphate; Glut, glucose transporter; GSIS, Glucose-stimulated insulin secretion; K^+^, Phosphate; ME1, Malic enzyme 1; mMDH, Mitochondria malate dehydrogenase; MPC, Mitochondrial pyruvate Carrier; NADH, Nicotinamide adenine dinucleotide + Hydrogen; NADPH, Nicotinamide adenine dinucleotide phosphate; NEFA, Non-esterified fatty acids; NOX, NADPH oxidase; O_2_^·−^, superoxide anion; OMC, oxoglutarate/malate carrier; OMT, 2-oxoglutarate/malate transporter; PC, Pyruvate carboxylase; PDH, Pyruvate dehydrogenase; Pi, Inorganic phosphate; PKC, protein kinase C.

**Figure 2:**
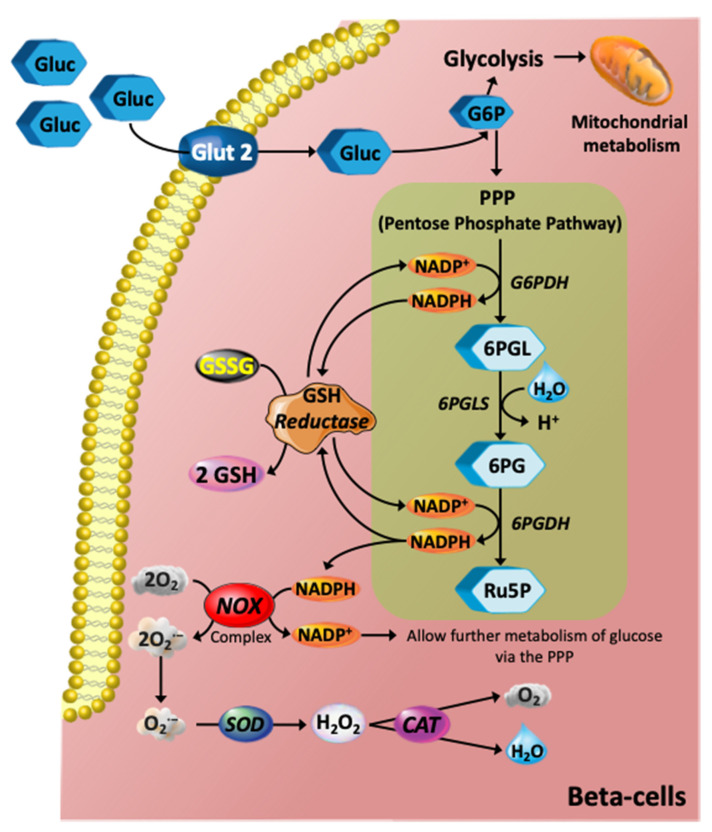
Glucose metabolism via the PPP and its role in redox pathways. The glycolytic intermediate glucose 6-phosphate (G6P) can be diverted into the PPP, which is an NADPH-producing pathway. In turn, NADPH is an electron donor for the *de novo* glutathione (GSH) system mediated by GSH reductase using glutathione disulfide (GSSG) to generate reduced glutathione (GSH). The GSH system is the most abundant intracellular non-protein thiol that exerts essential antioxidant roles against ROS in all animal cells. NADPH is also an important electron donor for the NADPH oxidase (NOX) complex, and hence further contributing to stimulation of glucose metabolism via PPP. The NOX complex, however, produces the superoxide anion (O_2_^·−^), which is an anion radical that can be rapidly converted to hydrogen peroxide (H_2_O_2_) by the action of superoxide dismutase (SOD) and further catalysed by catalase (CAT) producing oxygen and H_2_O. Abbreviations: 6PG, 6-phosphogluconate; 6PGDH. 6-phosphogluconate dehydrogenase; 6PGL, 6-Phosphogluconolactonase; 6PGLS, 6-phosphogluconolactonase; Ru5P, ribulose-5-phosphate.

The availability of NADPH, which is generally required for biosynthetic pathways such as fatty acid synthesis, and the regeneration of reduced glutathione, is central to antioxidant status in the β-cell. A large proportion of cellular production of NADPH comes from the pentose phosphate pathway (PPP) [28,29]. Generation of NADPH in high-glucose conditions can be considered protective, as the PPP diverts glucose away from possible ROS-generating processes (i.e., glycolysis and oxidative phosphorylation) and allows glucose to be utilised in alternative pathways. NADPH is absolutely required for converting oxidized glutathione (GSSG) into the reduced state so that it can act to remove ROS (Figure 2), possibly generated by the mitochondrial respiratory complexes, or from active plasma membrane NADPH oxidase (NOX) activity [30]. NOX can function to regenerate NADP^+^ from NADPH, thus promoting metabolism of glucose via the PPP, albeit producing O_2_^·−^ in the process, which must undergo conversion to H_2_O_2_ via superoxide dismutase, then subsequent conversion to H_2_O and O_2_ via catalase (Figure 2). This activation of the PPP is particularly important as β-cells have multiple options available for glucose metabolism, including glycolysis, PPP, and glycogen formation, all of which can impact acute or chronic level insulin secretion. Changes in glucose metabolism will certainly impact insulin secretion in T2DM and greater clarification of the pathways that are altered is now required.

### 2.2. Lipids

Pancreatic β-cells, exposed to both high glucose concentrations and saturated non-esterified fatty acids (NEFAs), are associated with a substantial increase of insulin release, but chronic exposure results in desensitization and reduction in secretion [31]. Lowering of plasma NEFA levels in fasted rats or humans impaired glucose-dependent insulin release. Thus, changes in plasma levels of NEFAs over physiological concentration ranges are essential for β-cell function. Although it is widely reported that intracellular NEFA metabolism (especially FA synthesis and formation of LC-acyl-CoA) is required for promotion of insulin secretion, the key regulatory molecular events linking glucose and fatty acid metabolism and thus insulin secretion are not well described. LC-acyl-CoA (derived from endogenous or exogenous LCFA) impacts several metabolic processes in the β-cell including activation of certain isoforms of PKC, modulation of ion channels, protein acylation, protein malonylation, thus leading to metabolic reprogramming by unknown mechanisms, ceramide- and/or NO-mediated apoptosis, generation of ROS and binding to and modulating nuclear transcriptional factors [30,32].

There is clearly a role for Triacylglycerol (TAG)/NEFA cycling in the pancreatic β-cell, as blocking the TAG/NEFA cycling dramatically reduces insulin secretion [33]. It is likely the NEFAs released from the TAG/NEFA cycle are not re-esterified (β-cells release glycerol generated by breakdown of TAG, which indirectly reveals the level of TAG hydrolysis) [34] but play key signalling roles in the stimulation of insulin secretion. It would be most interesting to determine the level of cycling occurring in islets from control and diabetic mice or rats.

Malonyl CoA, derived from Acetyl CoA (which itself is derived from Citrate via ATP-Citrate lyase) is formed in the ‘committed’ step of FA synthesis catalysed by Acetyl CoA Carboxylase, is a key metabolic coupling factor in stimulation of insulin secretion [35]. Malonyl-CoA is a signal that acts as a ‘metabolic switch’, playing an essential role in insulin secretion stimulated by glucose and other fuel stimuli, especially when islet lipid oxidation is increased [36]. Thus, the ACC/malonyl-CoA/CPT1 relationship is critical in GSIS due to their role in a metabolic on/off mechanism, in line with the significance of the glycerolipid/NEFA cycle and associated lipogenesis and lipolysis arms in GSIS (Figure 1). Indeed, Malonyl-CoA has been described as a ‘regulatory’ metabolic coupling factor, controlling insulin secretion at early steps in the amplification pathway [37]. The impact of metabolic adaption/reprogramming in type 2 diabetes on the latter metabolic on/off switching mechanism is not known.

## 3. Evidence for Immune Driven Metabolic Adaptions in β-Cells, with a Focus on Increased Oxidative and Endoplasmic Reticulum Stress

T1DM is a chronic autoimmune disease, which is often diagnosed in childhood. The mechanisms which drive β-cell death and dysfunction are immune-cell and immune-molecule related. In contrast, T2DM results from aberrant metabolism [38]. The contributions of various immune cells and immune factors to both Type 1 and Type 2 diabetes are likely to be significantly different, but there is considerable evidence for activation of the metabolic ROS/ER stress pathways in the pancreatic β-cell [16,17]. The impact of ROS/ER stress on metabolic adaptions in the β-cell have been previously reviewed [30,31] but further work is required to gain further insight in this area.

Islet inflammation in T1DM is characterized by leukocyte infiltrates [30], in particular macrophages and T-cells which damage β-cells by release of cytokines, ROS and NO and also activation of death-receptor-mediated death pathways and subsequent phagocytosis [30,32]. Production of cytokines such as INF-γ, TNFα and IL-1β act in synergy to promote elevation in concentration and increase in activity of NADPH oxidase and iNOS consequently increasing the formation of products including ROS and NO, respectively. The mechanism of action of INF-γ, TNFα and IL-1β involves stimulation of transcription factors including NFκB (in mouse islet β-cells) [30]. Generation of ROS may elevate ER stress and possibly promote cell death. Furthermore, activation of β-cell NFκB may result in autocrine production of cytokines, thus amplifying these death signals [38].

Associated with T1DM and associated immune-mediated reduction of β-cell mass is a reduction in islet insulin secretion capacity [39], which will lead to additional hyperglycaemia and dyslipidaemia in these patients [40]. The associated excess of glucose and circulating free fatty acids can elevate ROS production and ER stress resulting in a build-up of unfolded proteins in the ER [23,41]. Elevated ROS and ER stress may activate caspase enzymes via mitochondrial- and ER-mediated death pathways, respectively. ROS/RNS can also activate NFκB-dependent stress pathways, which may promote transcription of genes coding either cytokines or immune cell chemo-attractants leading to a destructive cycle of events [23,42].

A link between glucotoxicity and inflammatory processes has been suggested [42,43]. It has been reported that addition of IL-1Ra, the IL-1 receptor antagonist (Anakinra), protected islets from IL-1β, but also reduced blood glucose in a small clinical trial of T2DM patients [43,44,45].

Furthermore, it has been demonstrated that IL-1β, IFN-γ and TNF-α contribute to cytokine-mediated β-cell apoptosis by downregulating the sarcoplasmic/endoplasmic reticulum Ca^2+^ pump (SERCA2B), causing severe ER Ca^2+^ depletion and cytosolic Ca^2+^ calcium elevation [46,47,48,49]. This cytokine-induced disruption to intracellular Ca^2+^ not only leads to protein misfolding and activation of ER stress pathways, but can impact mitochondrial metabolism. Increases in cytosolic Ca^2+^ levels can increase mitochondrial Ca^2+^, which in turn depolarizes the mitochondrial membrane potential and thus reduces ATP production [50,51,52]. While able to act as a buffer for transient increases in Ca^2+^ levels, a sustained overload in mitochondrial Ca^2+^ results in dysfunction, impedes β-cell function and can even induce apoptosis [46,52,53].

## 4. Evidence for Endocrine Regulation of Metabolic Adaptions/Reprogramming in β-Cells

Secreted from the intestinal L- and K-cells, the incretin hormones gastric inhibitory polypeptide (GIP) and glucagon-like peptide-1 (GLP-1) play a key role in regulating glucose homeostasis [53,54]. Released postprandially, these incretin hormones act by binding to their associated receptors, the GLP-1R and GIPR. Once bound, these class B G protein-coupled receptors (GPCR) trigger a downstream signalling cascade able to potentiate GSIS in β-cells, as well as regulating glucagon release in α-cells [54,55,56]. In T2D patients, however, the insulinotropic response to GIP is substantially diminished, while the action of GLP-1, in contrast to GIP, are preserved [57,58]. Consequently, GLP-1 and its long lasting analogues are currently considered an effective therapy for T2D, and have been demonstrated to stimulate a range of anti-diabetic effects including: an increase in satiety; inhibition of food motivated ‘behaviour’ and a reduction in gastric emptying [59,60]. GIP, on the other hand, has not been extensively pursed as a treatment for T2D due to its impaired incretin effect in patients with T2D, coupled with its debated obesogenic propensity. Recently, however, the effectiveness of GIP as a diabetic therapeutic agent has been reappraised based on a growing body of new evidence indicating that promoting GIP action/activity, either alone or in combination with GLP-1 analogues, is beneficial for treating T2D [53,57]. While both GLP-1 and GIP have a plethora of extra-pancreatic actions, this section of the review outlines the current knowledge in regard to the metabolic impact of these incretins on pancreatic β-cell function and the physiological consequences.

Classically, the incretin-mediated effects of GLP-1 and GIP in the pancreatic β-cell arise in response to the downstream signalling cascade triggered by ligand binding. Once bound, the GLP-1R and GIPR undergo conformational changes initiating a cascade that results in the activation of membrane bound Adenyl Cyclase (AC) and consequent production of cyclic cAMP. The rapid increase in cAMP results in the direct activation of Protein kinase A (PKA) and ‘exchange protein directly activated by cAMP isoform 2′ (EPAC2) [57,60,61,62]. Activation of these cAMP effectors results in modification of various targets within the secretory machinery, which can act synergistically to enhance GSIS. Mechanisms regulated by these cAMP effectors can include: closure of ATP-sensitive K^+^ channels [61]; elevation of cytosolic Ca^2+^ levels [62]; stimulation of insulin granule mobilisation, priming and exocytosis; as well as inhibition of β-cell repolarization by reducing Kv channel currents [55,63]. Notably, recent studies have demonstrated that in addition to directly stimulating insulin secretion through the β-cell GIPR, GIP stimulates α-cell glucagon secretion to enhance α to β-cell communication, and thereby potentiate insulin secretion to a greater degree than GIPR activation in β-cells alone, but only at high glucose levels and in the presence of amino-acids [64,65]. Moreover, findings from these studies have demonstrated in both mouse and human islets that while GLP-1 and glucagon can activate their associated receptors in the β-cell, in the presence of glucose and amino acids, the potentiation of GSIS results predominantly from local glucagon activation of the GLP-1R [64,65]. These studies demonstrated that abrogated glucagon secretion results in markedly impaired insulin secretion and glucose intolerance, and in the fed state complements the actions of insulin, rather than oppose, in order to maintain euglycemia [64,66]. Further challenging the classical endocrine model of GLP-1 action, recent research has demonstrated that pancreatic derived GLP-1 is necessary for glucose homeostasis, whilst intestinally derived GLP-1 is dispensable [67,68]. Findings from these studies demonstrated that pancreatic specific expression of the preproglucagon (Gcg) gene, and thus GLP-1 production, was required for glucose regulation, whereas intestinal specific Gcg expression did not make an important contribution [67,68]. Coupled with the knowledge that α-cells isolated from human and mouse islets contain the necessary components to secrete GLP-1 [69], these recent findings support the emerging concept of a paracrine model wherein insulin secretion is regulated by α-cell derived GLP-1 binding to the GLP-1R of neighbouring cells [68,69,70,71].

In addition to potentiating acute GSIS, activation of the GLP-1R and GIPR signalling cascades can promote insulin gene transcription, induce pro-survival and anti-apoptotic responses, stimulate DNA synthesis, as well as promote metabolic reprogramming [60,61,72]. These beneficial effects are believed to stem from incretin induced non-receptor tyrosine kinase/c-Src transactivation of epidermal growth factor receptors (EGFRs) [70], as well as cAMP-mediated signalling and thus ultimately, activation of pro-survival signalling through cAMP responsive element binding (CREB) [59,71]. Although the incretins and their receptors are highly related, they can act through divergent downstream signalling pathways to exert their effects (reviewed in [55,60]). Indeed, in mouse islets GIP, but not GLP-1, has been demonstrated to control T cell-specific transcription factor-1 expression [72,73], while GLP-1R signalling but not GIPR signalling, is able to increase the expression levels of insulin receptor substrate 2 (*Irs2*), *Egfr*, and Glucokinase (*Gck*) [70,74]. As both IRS-2 and EGFR signal transduction pathways induce pro-survival and anti-apoptotic responses, this may be a contributing factor to the increased sensitivity to streptozotocin-induced apoptotic injury seen in β-cells lacking a GLP-1R, but not a GIPR [75,76]. Additionally, both GIP and GLP-1 have anti-apoptotic, and anti-oxidant, actions involving activation of the CREB and AKT pathways, promoting phosphorylation, and thus nuclear exclusion of, the nuclear transcription factor Foxo1, and leading to the up-regulation of the anti-apoptotic and down-regulation of the pro-apoptotic genes *bcl-2*, and *bax*, respectively [77,78]. However, while GLP-1R agonism has been demonstrated to induce AKT activation in a PI3K/PKA-dependent mechanism [79,80], GIPR activation has been proposed to exhibit anti-apoptotic, as well as anti-oxidant actions, through dual suppression of p38 MAPK and JNK, phosphorylation of AKT (473) independent of PI3K/PKA activation, and inhibition of apoptosis signal regulating kinase-1 (ASK1) activation, which has been demonstrated to act as a redox sensor upon exposure to excessive levels of ROS [77,81]. Notably, while GIP has been reported to reduce oxidative stress via ASK1 suppression, chronic GLP-1R agonism has been reported to enhance GSH content, glutathione reductase (GR) activity, expression of glutathione peroxidase (GPx) and catalase (*Cat*), as well as increase the protein levels and translocation of the nuclear transcription factor erythroid 2p45-related factor (Nrf2), through activation of the cAMP/PKA/ERK pathway in in vitro and in vivo [82,83,84,85]. Recent reports have described the effects of addition of the GLP-1 receptor antagonist (Liraglutide) on pancreatic β-cell apoptosis in diabetes. The NADPH oxidase isoform NOX2 levels were reduced in high glucose exposed β-cells, on Liraglutide treatment, via increase in phosphorylation of AMPKα which prevented NOX2 activation and subsequently reduced apoptosis [86]. In addition, cAMP inducers have been reported to suppress glucose-induced ROS production similarly to NOX2 deficiency, enhancing insulin secretion [87].

Furthermore, both GIP and GLP-1R signalling have been reported to promote β-cell replication through the transcriptional induction of cyclin D1, as well as the Raf-Mek1/2- extracellular signal-regulated kinase 1/2 (ERK1/2) signalling pathway [88,89]. A critical difference, however, is that GLP-1R activation, but not GIPR, is able to regulate PDX1 expression [90], induce expression of IRS2 [74], as well as stimulate an IGF-1R/IGF-2 autocrine loop that not only enhances β-cell proliferation, but also promotes cell survival [91,92]. Of note, it has been demonstrated that chronic GLP-1R signalling induces biphasic cAMP-dependent gene expression in β-cells. While the first wave is CREB-mediated, occurring within 2 h, the second phase, at 16 h, is orchestrated by mTOR induction of hypoxia-inducible factor (HIF), and the accumulation of its α-subunit (HIF-1α). A heterodimeric transcriptional factor composed of two subunits, HIF induces metabolic reprogramming in response to growth factor signalling, and hypoxia [93,94]. Extending these findings, Carlessi and colleagues recently reported that chronic GLP-1R stimulation leads to metabolic reprogramming, characterised by increased glycolysis, ATP production, enhanced GSIS, and upregulation of glycolytic enzymes, through the GLP-1/mTOR/HIF-1α signalling pathway. The authors also demonstrated that the GLP-1R-mediated metabolic reprogramming was dependent on PKA, PI3K and EPAC2 signalling, but not the IGF-2/IGF1R autocrine loop [95,96]. It has previously been reported that in β-cells, chronic changes in the rate of glycolysis are able to increase β-cell proliferation independent of actual circulating glucose levels [97,98]. Furthermore, it has been demonstrated that the mitogenic effect of glucose metabolism is dependent on changes in both glycolysis and membrane depolarization [98]. Coupling these data with the findings that chronic GLP-1R signalling promotes β-cell glucose metabolism via mTOR/HIF-1α activation and membrane depolarization, offers an additional molecular mechanism by which GLP-1R induced metabolic reprogramming may act to boost β-cell mass, increase insulin section, reduce ROS generation, and ultimately restore euglycemia. While chronic GIPR stimulation has previously been reported to induce cAMP and AKT/PKB signalling in studies assessing β-cell survival [99,100], the impact of chronic GIPR signalling on metabolic reprogramming has yet to be evaluated. It would therefore be useful for future studies to elucidate the effect of chronic GIPR signalling on metabolic reprogramming, and thus β-cell function (including insulin secretion) and survival. Taken together, these data highlight the diverse and divergent metabolic adaptations of β-cells induced by incretins and offers new avenues for future research.

## 5. Evidence for Pharmaceutical Drug Induced Metabolic Adaptions/Reprogramming in β-Cells

### 5.1. Antidiabetic Medications and Pancreatic β-Cell Metabolism

Recent studies indicate that metformin, GLP-1R agonists and the dipeptidyl peptidase-4 (DPP-4) inhibitors have direct effects on β-cells, promoting insulin secretion and/or limiting β-cell damage [101]. Metformin has been used therapeutically since the 1950s, is still the first-line treatment for T2DM as an effective antidiabetic agent, by reducing hepatic glucose production [101], restoring insulin secretion and protecting pancreatic β cells from lipotoxicity or glucotoxicity [102]. Metformin primarily acts on insulin-sensitive tissues. Observations from diabetic animal models and clinical trials have reported metformin can inhibit gluconeogenesis and suppress hepatic glucose production with improved insulin sensitivity in all peripheral tissues except the brain [103]. Metformin has pleiotropic actions in multiple organs or systematically [104] and can also partially ameliorate pancreatic β cell failure that occurs in T2DM [105].

Under homeostatic conditions, metformin does not promote or inhibit insulin secretion [106]; however, it reduces fasting plasma insulin concentration and enhances insulin sensitivity through reducing the activity of the pathway of hepatic gluconeogenesis, with a lesser effect on glycogenolysis [107]. Metformin directly modulates pancreatic β-cell function including moderate increase in insulin release, transcriptional regulation of β-cell signalling and cell viability, which is dependent on the presence of glucose [108], [109]. Metformin can restore insulin secretion previously suppressed by free fatty acids or high glucose in both human islets [110,111] and cell lines [105,112] due to improvements in glucose metabolism in the β-cell [113]. In experiments using Human islets cultured in high glucose concentrations, metformin reversed a reduction in GSIS associated with reduced ATP levels and a lower ATP/ADP ratio. Furthermore, metformin inhibited the activity of mitochondrial complex I and reversed ultrastructural alterations in β-cells exposed to high glucose levels [113,114]. Metformin prevented Ca^2+^-induced PTP opening in permeabilized and intact INS-1 cells to preserve β cell viability in hyperglycaemic conditions [115].

It has also been reported that metformin inhibits thapsigargin induced ER stress-induced apoptosis in a mouse pancreatic β cell line (NIT-1 cells) via upregulation of AMPK-PI3 kinase-JNK pathway [116]. Similarly, metformin protects rat insulinoma INS-1 cells from palmitate induced lipotoxic ER stress and apoptosis through decreasing JNK and p38MAPK phosphorylation via MAPK signalling pathways [117,118]. Moreover, metformin upregulates Aquaporin 7 in pancreatic islets (INS-1 cells) damaged by hyperglycaemic conditions through suppression of p38 and JNK mitogen activated protein kinase signalling to promote glycerol influx into β cells and subsequent promotion of insulin secretion [119].

Metformin prevented glucotoxicity through the reduction of oxidative and ER stress determined by a reduction in CD36 expression in pancreatic β-cells [120]. Pancreatic islets have relatively low expression of antioxidant enzymes which increases their risk of oxidative stress, see Section 2, Section 3 and Section 4 above [121]. Incubation of primary rat islets with metformin has been reported to restore insulin and pancreatic duodenal homeobox1 (Pdx) 1mRNA expression with recovery of GSIS and decreases in ROS production. Pancreatic duodenal homeobox -1 is a transcription factor that is expressed in β-cells and in duct cells of the pancreas stimulating cell proliferation and differentiation to form new islets and contributes to β-cell mass expansion and glucose metabolism induced via Akt signalling. [122,123]. Metformin also improved the antioxidant status (superoxide dismutase and catalase), glucose homeostasis and reduced inflammatory (TNF-α, IL-6) and ER stress (ATF4) markers in the pancreas of diabetic rats [124]. Furthermore, metformin increases gene expression in INS-1 β cells and mouse islet cells of GLP-1R, GIPR, and G protein-coupled receptor 40 (GPR40), and peroxisome-proliferator activated receptor α (PPARα) [125,126,127].

It is well established that failure of monotherapy can occur with metformin use in clinical practice [128]. Metformin was compared to rosiglitisone and glyburide (sulphonylurea) for control of hyperglycaemia, assessed over 5 years to determine the durability of each monotherapy [129]. β-cell function declined over 6-months in all three groups with the annual rate of decline highest in the glyburide group (a decrease of 6.1%), intermediate in the metformin group (a decrease of 3.1%), and least in the rosiglitazone group (a decrease of 2.0%). Metformin is currently prescribed when 3 months of lifestyle and dietary interventions have not reduced hyperglycaemia; however, as perturbations in β-cell function are already causing impaired glucose tolerance, it has been suggested that metformin therapy should be implemented earlier with lifestyle interventions to preserve β-cell function [130]. When monotherapy fails to control hyperglycaemia, agents with different pharmacological actions are combined to control blood glucose levels in T2DM patients and prevent chronic hyperglycaemia and the development of comorbid macro and microvascular diseases. In contrast, combinations of medications should be considered with respect to optimising β-cell metabolism and ultimately function, particularly during gluco- and lipotoxic conditions.

The second class of drugs that has historically been prescribed with metformin are the sulphonylureas, which bind to the ATP-sensitive potassium channels (K_ATP_) to depolarise pancreatic β-cells and stimulate insulin release (see Marrano [101] for review of anti-diabetic drugs). Long-term exposure of the β-cells to sulphonylureas, both in in vitro and in vivo experiments, has been reported to decrease the extent of stimulated release of insulin due to desensitisation to this class of antidiabetic agent. For example, prolonged treatment of rodent BRIN-BD11 cells with glibenclamide reduced the acute insulinotropic actions of glucose [131]. Glibenclamide has also been demonstrated to increase apoptosis in human pancreatic islet cells [132]. The newer classes of antidiabetic agents include the thiazolidinediones which are peroxisome proliferator activated gamma receptor (PPARγ) activators that stimulate intracellular production of mediators that improve insulin sensitivity in tissues and the sodium-glucose transport protein 2 (SGLT2) inhibitors prevent renal absorption of glucose to increase excretion of glucose and reduce hyperglycaemia in patients with T2DM. Both of these classes of anti-diabetic agents can indirectly protect β-cell function through reducing hyperglycaemia. Dapagliflozin, a SGLT2 inhibitor, has been reported to decrease blood glucose concentrations and through upregulation of insulin and GLP-1 levels in T2DM mice, exhibit protective effects on the β-cells and enhanced β-cell replication [133].

Finally, GLP-1 receptors agonists (as previously discussed) and the DPP-4 inhibitors such as sitagliptin which inhibit breakdown of GLP-1 and GIP to elevate plasma levels of these hormones to stimulate insulin secretion and inhibit glucagon release, have the capacity to directly modify metabolic activity in β-cells. DPP-4 inhibition, through elevations in GLP-1, has been shown to activate CREB in insulin positive β-cells leading to increased Bcl-2, BIRC3 levels to inhibit apoptosis and elevate IRS-2, an adapter protein involved in insulin signalling [134].

### 5.2. Pancreatic β-Cell G-Protein Coupled Receptors and Cell Metabolism

Many β-cell G-protein coupled receptors need to be explored for their capacity to modulate insulin secretion [135]. Well established examples of islet GPCR are the receptors for the classical autonomic nervous system neurotransmitters such as acetylcholine (M_3_ muscarinic receptors) and noradrenaline (β_2_- and α_2_-adrenoceptors) which modulate insulin release under different conditions. Islet GPCRs that are sensitive to fatty acid ligands include the GPR40 and GPR119 and the receptors for the incretin hormones include GLP-1 and GIP as previously discussed. Islet GPCR for neuropeptides includes pituitary adenylate cyclase-activating polypeptide (PACAP), vasoactive intestinal polypeptide (VIP; PAC1 and VPAC2 receptors), cholecystokinin (CCKA receptors) and neuropeptide Y (NPY Y1 receptors). Other islet GPCR include cannabinoid receptors (CB_1_ receptors), vasopressin receptors (V1_B_ receptors) and the purinergic receptors (P_2Y_ receptors).

GPCRs in pancreatic β-cells are coupled to several signalling cascades including the cAMP/PKA/EPAC, and the inositol triphosphate (IP3)/diacylglycerol (DAG) pathways as well as changes to protein phosphorylation and protein acylation. In pancreatic β-cells, Gαs mediates increases in intracellular cAMP associated with increased insulin secretion, while Gαi mediates decreases in intracellular cAMP and inhibition of insulin secretion. Gαq mediates increases in IP3 and DAG production through the activation of phospholipase C (PLC) associated with increased release of Ca^2+^ from the ER and enhanced insulin secretion [136,137]. The M_3_ muscarinic receptors are functionally expressed in β-cells and enhance insulin secretion when blood glucose levels are elevated via coupling to Gαq activated PKC which raises intracellular Ca^2+^ levels. A positive allosteric modulator of M_3_ muscarinic receptors has been shown to restore glucose homeostasis in diabetic, glucose intolerant mice by promoting insulin secretion and may be a target for future clinical treatment of T2DM [138]. The long-chain fatty acid receptor GPR40 plays an important role in potentiation of GSIS from pancreatic β-cells. Activation of GPR40 by long-chain free fatty acids such as palmitate or oleic acid increases Ca^2+^ release from the ER by activating inositol 1,4,5-triphosphate (IP3) receptors [139]. Indeed, elevated Ca^2+^ levels can stimulate metabolism mainly though activation of mitochondrial TCA cycle activity and subsequent elevation in ATP levels

G-protein coupled receptor-55 (GPR55), a novel endocannabinoid receptor [140], is activated by phytochemicals, endocannabinoids and small synthetic cannabinoids [140,141,142]. In the pancreas, both human and rodent islets express GPR55 as indicated from gene and protein expression studies [142,143,144]. Furthermore, immunohistochemical analysis of rat and mouse pancreas sections demonstrated GPR55 expression specifically in insulin-secreting β-cells, but not in glucagon- or somatostatin-releasing α- and δ-cells, respectively [142,143], and have been identified as an anti-diabetic target [141,145]. The metabolic functionality of GPR55 ligands was investigated using CRISPR/Cas9 gene editing to determine their regulatory role in β cell and incretin-secreting enteroendocrine cell function. Atypical and endogenous endocannabinoid ligands stimulate insulin secretion in rodent (BRIN-BD11) and human (1.1B4) β cells through upregulation of insulin mRNA. As demonstrated in a clonal GPR55 knockout β cell line, GPR55 agonists enhance insulin secretion in β -cells, GIP and GLP-1 release from incretin-secreting enteroendocrine cells [146]. The GPR55 is specific for cannabinoid endogenous agonists (endocannabinoids) and non-cannabinoid fatty acids including L-α-lysophosphatidylinositol [147], and couples to Gα_12/13_ and Gα_q_ proteins, leading to enhancing intracellular Ca^2+^, ERK1/2 phosphorylation and Rho kinase [140]. Primary studies have shown that activation of GPR55 receptors in pancreatic β-cells augments intracellular Ca^2+^ release, indicating a role for IP3 and intracellular calcium stores through PLCβ signalling [145,147,148,149,150,151]. Activation of the GPR55 also upregulates antiapoptotic genes such as Bcl-2 and Bcl-xL, reduces ER stress-mediated apoptosis and activation of the transcription factor cAMP response element binding to promote β-cell survival [146,148].

### 5.3. Contribution of Insulin, and Therapeutic Availability, to Metabolic Function of β-Cells

β-cells express insulin receptors, and autocrine insulin signalling is important for maintaining β-cell function by regulating cell proliferation, apoptosis and gene transcription [149]; however, the evidence is conflicting as to whether insulin mediates positive feedback of its own secretion in human islets [101]. Insulin increases the cytosolic Ca^2+^ concentration ([Ca^2+^]i) in rodent and human β-cells [150,151,152] by releasing Ca^2+^ from thapsigargin- and Nicotinic acid adenine dinucleotide phosphate (NAADP)-sensitive stores [151] but with minimal effects on membrane potential. Inhibition of insulin receptors reduces insulin secretion induced by other secretagogues, demonstrating that the β cell insulin receptor can provoke positive feedback for insulin secretion [150]. Increased [Ca^2+^]_i_ was not accompanied by a stimulation of insulin release; therefore, IR-mediated signalling may affect other processes that regulate intracellular insulin release levels. In contrast, insulin (at concentrations comparable to those measured in plasma) has been found to inhibit glucose-induced Ca^2+^ oscillations in mouse islet cells and activate K_ATP_ channels to cause membrane hyperpolarization, suggesting that locally released insulin might generate a negative feedback signal within the islet during basal conditions. [153]. As a possible underlying mechanism, it was reported that insulin acutely activates PI3 kinase and increases PIP_3_ formation in an autocrine manner [154]. Acute disruption of insulin signalling using the tyrosine kinase inhibitor genistein in mouse-isolated islets [155] or the PI3 kinase inhibitor wortmannin in rat-isolated perfused islets [156] has also been reported to potentiate GSIS. As stated above, elevated Ca^2+^ levels can stimulate metabolism mainly though activation of mitochondrial TCA cycle (Ca^2+^ sensitive) enzyme activity and subsequent elevation in ATP levels.
